# Highly Flexible and Transparent Ag Nanowire Electrode Encapsulated with Ultra-Thin Al_2_O_3_: Thermal, Ambient, and Mechanical Stabilities

**DOI:** 10.1038/srep41336

**Published:** 2017-01-27

**Authors:** Byungil Hwang, Youngseo An, Hyangsook Lee, Eunha Lee, Stefan Becker, Yong-Hoon Kim, Hyoungsub Kim

**Affiliations:** 1 BASF Electronic Materials R&D Center Asia, Suwon, 16419, Republic of Korea; 2 School of Advanced Materials Science and Engineering, Sungkyunkwan University, Suwon, 16419, Republic of Korea; 3 Analytical Engineering Group, Samsung Advanced Institute of Technology (SAIT), Samsung Electronics Co., Suwon 16678, Republic of Korea; 4 SKKU Advanced Institute of Nanotechnology (SAINT), Sungkyunkwan University, Suwon, 16419, Republic of Korea

## Abstract

There is an increasing demand in the flexible electronics industry for highly robust flexible/transparent conductors that can withstand high temperatures and corrosive environments. In this work, outstanding thermal and ambient stability is demonstrated for a highly transparent Ag nanowire electrode with a low electrical resistivity, by encapsulating it with an ultra-thin Al_2_O_3_ film (around 5.3 nm) via low-temperature (100 °C) atomic layer deposition. The Al_2_O_3_-encapsulated Ag nanowire (Al_2_O_3_/Ag) electrodes are stable even after annealing at 380 °C for 100 min and maintain their electrical and optical properties. The Al_2_O_3_ encapsulation layer also effectively blocks the permeation of H_2_O molecules and thereby enhances the ambient stability to greater than 1,080 h in an atmosphere with a relative humidity of 85% at 85 °C. Results from the cyclic bending test of up to 500,000 cycles (under an effective strain of 2.5%) confirm that the Al_2_O_3_/Ag nanowire electrode has a superior mechanical reliability to that of the conventional indium tin oxide film electrode. Moreover, the Al_2_O_3_ encapsulation significantly improves the mechanical durability of the Ag nanowire electrode, as confirmed by performing wiping tests using isopropyl alcohol.

With increasing demand for flexible/stretchable electronics, much effort has been devoted to the development of transparent conductors with low resistance and high flexibility that can withstand external influences such as high temperatures and corrosive environment^1^,^2^,^3^,^4^,^5^,^6^. Among the many possible candidates for the replacement of the conventional indium tin oxide (ITO) electrode that has several limitations, namely, brittleness and high processing cost^7^,^8^, Ag nanowire-based electrodes are the most promising due to the low sheet resistance, high flexibility/stretchability combined with high optical transmittance in the visible range^9^,^10^,^11^,^12^,^13^. However, a major drawback is its poor thermal and ambient stability demonstrated by the phenomenon of agglomeration at a much lower temperature (~200 °C) than its melting point and rapid oxidation when exposed to humid air^1^,^6^,^10^. Since the Ag nanowire electrode may experience thermal and oxidative stresses during post-manufacturing processing as well as during actual operation, the development of highly reliable transparent electrodes from Ag nanowires with improved thermal and ambient resistance is urgently needed.

To resolve the issues concerning thermal and ambient stability, several studies have proposed the encapsulation of the Ag nanowire electrode by a material with a high melting temperature^1^,^2^,^3^,^14^,^15^,^16^. For example, Song *et al*. used a sol-gel-deposited TiO_2_ film as a capping layer^1^, and Kim *et al*. introduced a sandwich-like structure where the Ag nanowire electrode was positioned between two sputtered ZnO layers^3^. Recently, a couple of research groups adopted a low-temperature atomic layer deposition (ALD) process to coat conformal encapsulation layers, such as ZnO and Al_2_O_3_, on Ag nanowires^14^,^15^,^16^. Although these previous works are significant, the thermal stability (up to 300 °C) reported for coated Ag nanowires is not sufficient when applied to a typical device manufacturing process such as low-temperature poly-silicon fabrication that requires a thermal resistance to withstand temperatures more than 350 °C and a high ambient resistance to be stable for more than 500 h in humid air^17^,^18^. Moreover, the ambient and mechanical robustness of the Ag nanowire-based electrodes have not been thoroughly analyzed or discussed in detail to validate the use of Ag nanowire electrodes in practical electronic applications. For the previous approach using the Al_2_O_3_ encapsulation layer, only the successful ALD coating at a low temperature (50 °C) was demonstrated without characterizing the thermal, ambient, and mechanical stabilities of the Ag nanowire electrodes^14^.

In the present work, we report the detailed characterization results of a highly robust Ag nanowire transparent electrode encapsulated using an ultrathin Al_2_O_3_ layer (Al_2_O_3_/Ag nanowire electrode) fabricated by a simple low-temperature ALD process, especially focusing on its thermal, ambient, and mechanical stabilities. A more detailed comparison with previous works can be found in Table S1 (Supporting Information). It was revealed that the Al_2_O_3_/Ag nanowire electrode was able to withstand high temperatures up to 380 °C and was stable against oxidative stress for more than 1,000 h in humid air (a temperature of 85 °C and a relative humidity of 85%). In addition, the Al_2_O_3_/Ag nanowire electrode was also mechanically robust and stable under conditions of severe bending fatigue tests and wiping tests using isopropyl alcohol (IPA), which demonstrates its applicability in flexible and stretchable electronics experiencing harsh fabrication or operation conditions.

## Results and Discussion

### Optical and Electrical Properties

**Figure 1:**
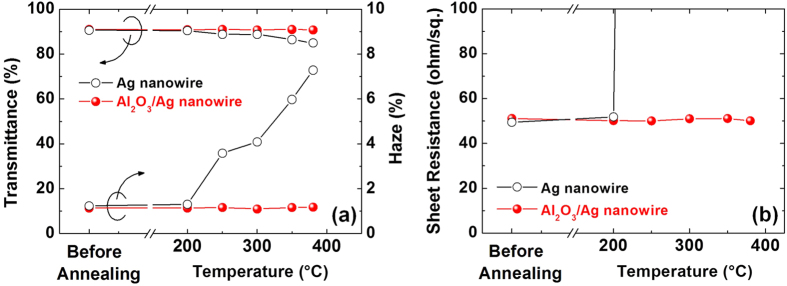
Changes in (**a**) optical transmittance/haze and (**b**) sheet resistance of the Ag and Al_2_O_3_/Ag nanowire electrodes as a function of the annealing temperature. The annealing time is 20 min.

Ag nanowire electrodes with excellent optical and electrical properties were fabricated using a simple and cost-effective doctor-blade method. As shown in Fig. 1, the transmittance, haze, and sheet resistance of the as-coated Ag nanowire electrodes on a glass substrate were 90 ± 3%, 1.3 ± 0.2% and 50 ± 3 ohm/sq., respectively. These values are comparable to those of sputtered indium tin oxide (ITO) thin films, which are commonly used as transparent electrodes^9^,^10^. Even after encapsulation with an ALD-Al_2_O_3_ layer, the Ag nanowire electrode retained its good optical and electrical properties with minimum change (<2%) in transmittance, haze, and sheet resistance values. This indicates that the Al_2_O_3_ coating carried out at a low ALD temperature of 100 °C did not significantly alter the optical/electrical properties of the Ag nanowire itself and also the interface between the two materials. Furthermore, the ultra-low thickness of the ALD-Al_2_O_3_ layer (~5.3 nm; the thickness was verified from transmission electron microscopy (TEM) images as shown in Figure S1 in the Supporting information) was responsible for the high optical transmittance obtained together with a high conductivity.

### Thermal Stability

**Figure 2:**
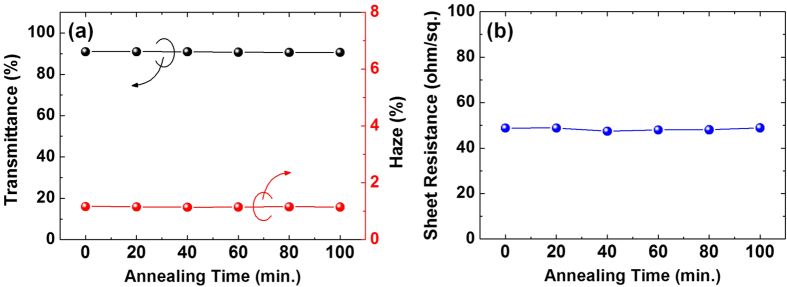
Changes in (**a**) optical transmittance/haze and (**b**) sheet resistance of the Al_2_O_3_/Ag nanowire electrode after annealing at 380 °C as a function of the annealing time.

To evaluate the thermal stability, the Ag nanowire electrodes with and without an Al_2_O_3_ encapsulation layer were annealed in air at different temperatures up to 380 °C for 20 min. Figure 1 shows the changes in transmittance, haze, and sheet resistance of the bare Ag and the Al_2_O_3_/Ag nanowire electrodes on the glass substrates after annealing at different temperatures. Both electrodes were stable up to 200 °C without degradation of the optical properties and did not show conductivity loss. However, as the annealing temperature was further increased, the optical and electrical properties of the bare Ag nanowire electrode degraded significantly. After annealing at 380 °C, the transmittance decreased from 90.7% to 85.0% and haze increased from 1.24% to 7.29%; the value for sheet resistance is not reported since it was higher than the measurement range of our equipment. In contrast, the Al_2_O_3_/Ag nanowire electrode showed negligible change in both optical and electrical properties after annealing at 380 °C for 20 min. In addition, this electrode also had high optical transmittance and electrical conductivity and showed a less than 1% change even after annealing at 380 °C for over 100 min, as shown in Fig. 2.

The above results indicate that the improvement of the thermal stability of Ag nanowires stems from the ultra-thin Al_2_O_3_ coating. Al_2_O_3_ has a high melting temperature (2070 °C)^19^ and even nanoscale alumina can withstand high annealing temperatures^20^,^21^. Furthermore, it has been reported that the trimethyl-aluminum (TMA) precursor of ALD-Al_2_O_3_ adsorbs strongly on Ag, which results in the conformal Al_2_O_3_ encapsulation of the Ag nanowires^22^,^23^. Since the surface diffusion of atoms is accelerated in a nanowire in view of its high surface-to-volume ratio, the Ag nanowire becomes unstable under thermal stress even at temperatures much lower than the melting point of bulk Ag^10^,^24^. However, the encapsulation with an ultra-thin Al_2_O_3_ layer effectively suppresses the surface diffusion of Ag atoms, thereby enhancing the thermal stability of Ag nanowires.

**Figure 3:**
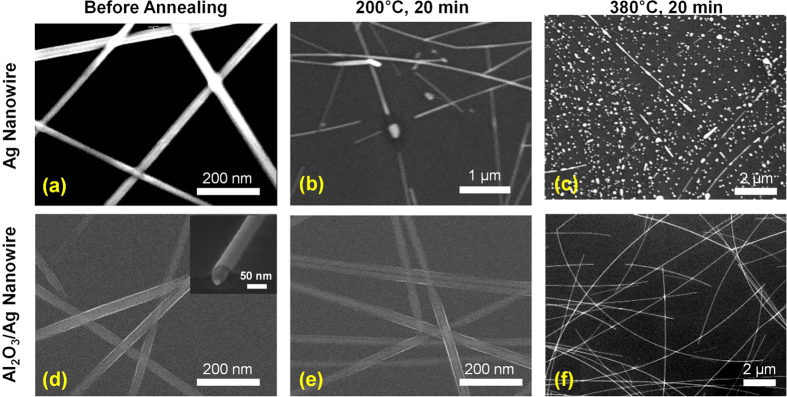
Plan-view SEM images of (**a**–**c**) bare Ag and (**d**–**f**) Al_2_O_3_/Ag nanowire electrodes after annealing at different temperatures. Inset of (**d**) is a tilted SEM image showing the conformal coating of an ultra-thin ALD-Al_2_O_3_ layer on a single Ag nanowire.

To further investigate the origin of the improved thermal stability, scanning electron microscopy (SEM) images were taken for the Ag and Al_2_O_3_/Ag nanowire electrodes annealed at different temperatures; these are presented in Fig. 3. The SEM images were in accordance with the measured changes in both electrical and optical properties for the different annealing temperatures (Fig. 1). The increase in the sheet resistance of the bare Ag nanowire after annealing at temperatures >200 °C was identified to be due to the loss of connectivity between the nanowires, as shown in Fig. 3b. This connectivity loss increased greatly as the annealing temperature was further increased to 380 °C, when most of the Ag nanowires agglomerated into a ball and the SEM image showed only dispersed Ag particles (Fig. 3c). The increase in the number of Ag particles can enhance light scattering from their reflective surfaces resulting in a dramatic increase in haze for the bare Ag nanowire electrode when annealed at temperatures >250 °C. On the other hand, the Al_2_O_3_/Ag nanowire electrode showed no such disconnection of junctions or rolling up of nanowires into balls even after annealing at 380 °C, as shown in Fig. 3f. The enhanced thermal stability is attributed to the conformal coating of ALD-Al_2_O_3_ on Ag nanowires. Although some amount of the polymeric residues from the Ag nanowire ink remained beneath the Ag nanowire, SEM (inset of Fig. 3d) and TEM analyses (Figure S1 in the Supporting Information) confirmed that the ultra-thin Al_2_O_3_ blanket film covered the Ag nanowire and the glass substrate seamlessly. Therefore, it is confirmed that the encapsulation by ultra-thin Al_2_O_3_ layer successfully prevents the Ag nanowires from thermal failure even when annealed at a significantly high temperature (380 °C) for a considerable duration time (100 min). Considering the actual manufacturing processes for display and electronics involving a post-annealing step for transistors where the devices are exposed to high temperatures for relatively long time periods (typically >1 h), it is expected that the Al_2_O_3_/Ag nanowire electrode can be successfully employed as a transparent electrode in view of its high thermal stability.

### Ambient Stability

**Figure 4:**
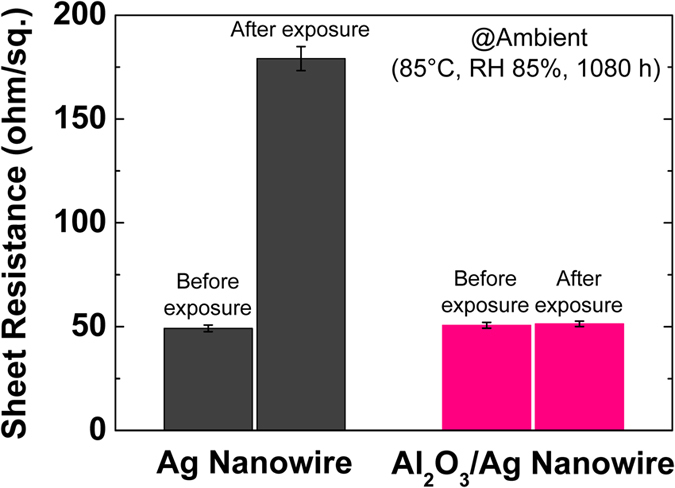
Sheet resistances of the Ag and Al_2_O_3_/Ag nanowire electrodes before and after exposure to ambient conditions with a relative humidity of 85% at 85 °C for 1,080 h.

A Ag nanowire is easily oxidized when exposed to humid air due to its large surface-to-volume ratio, which causes a significant degradation of its electrical conductivity^2^,^25^. However, the Al_2_O_3_ encapsulation layer, due to its low H_2_O permeability^26^,^27^, can prevent H_2_O molecules present in ambient air to contact with the embedded Ag nanowire. To evaluate the ambient stability, the bare Ag and Al_2_O_3_/Ag nanowire electrodes on the glass substrates were exposed to an air with a constant relative humidity of 85% at 85 °C for 1,080 h. Figure 4 shows the change in sheet resistance of both the nanowire electrodes before and after exposure to humid air. The sheet resistance of the bare Ag nanowire electrode dramatically increased from 49.2 to 179.1 ohm/sq. after exposure to humid air for 1,080 h, which could be due to the reduced cross-sectional area of Ag nanowires following surface oxidation^2^. In contrast, no increase in the sheet resistance was observed even after a continuous exposure to humid air for 1,080 h, which confirmed that the Ag nanowires were well protected from surface oxidation by the conformal Al_2_O_3_ encapsulation layer even when the thickness of this layer is very small (~5.3 nm).

### Mechanical Stability

In view of its network structure that gives it an advantageous geometry and limited dislocation activity in a nanoscale volume, a Ag nanowire electrode is known to show excellent mechanical reliability while responding to an external bending stress^28^,^29^. The nanowire network structure can accommodate the applied bending stress without heavily straining the individual nanowires, thereby enhancing the mechanical reliability under bending stress^28^,^29^. Moreover, the lack of dislocation activity in a nanoscale volume due to the dislocation starvation effect can strengthen the nanowire and prevent the accumulation of dislocations within the nanowire^30^,^31^,^32^, all of which lead to an excellent mechanical reliability for the Ag nanowire.

**Figure 5:**
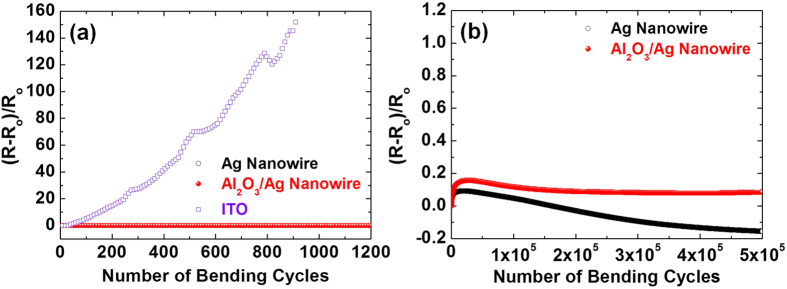
(**a**) Fractional resistance changes of the Ag and Al_2_O_3_/Ag nanowire electrodes under 2.5% applied strain as a function of the number of bending cycles: up to (**a**) 1,200 and (**b**) 500,000 cycles. The corresponding value for the reference ITO sample is also included in (**a**).

To examine the effect of Al_2_O_3_ encapsulation on the mechanical reliability of a Ag nanowire electrode, a cyclic bending test was conducted using a bending fatigue tester. The bending fatigue tester is capable of applying a bending stress for more than 500,000 cycles while *in situ* monitoring the change in the sample resistance^28^,^33^. The imposed bending strain for the test was set at 2.5%, which corresponds to a bending radius of 2.5 mm for a given substrate thickness of 125 μm. Figure 5a shows the fractional change in the resistance of the Ag nanowire electrodes with and without Al_2_O_3_ encapsulation compared to that of an ITO electrode (with a thickness of ~100 nm and a sheet resistance of ~50 ohm/sq.), as a function of the number of bending cycles. All the electrodes were prepared on the flexible polyethylene terephthalate (PET) substrates. The cyclic bending test result indicates that the Ag and Al_2_O_3_/Ag nanowire electrodes have excellent mechanical reliability as compared to the conventional ITO electrode. While the fractional resistance of the ITO electrode showed a drastic increase, the maximum increase during the 500,000 cycles of bending for the Al_2_O_3_/Ag nanowire electrode was only 15.9%, as shown in Fig. 5b. The ultra-thinness and the conformal nature of the Al_2_O_3_ encapsulation layer as enabled by ALD gives it the capability to bear more bending strain, thereby making the Al_2_O_3_/Ag nanowire electrode more flexible.

Meanwhile, it was observed that the mechanical reliability of Al_2_O_3_/Ag nanowire electrode was somewhat inferior to that of the bare Ag nanowire electrode that showed a lower maximum value of the fractional resistance increase during the early stage of a cyclic bending test, as shown in Fig. 5b. During the bending of an Al_2_O_3_/Ag nanowire electrode, cracks can be initiated and propagated from the Al_2_O_3_ encapsulation layer due to the brittle nature of the Al_2_O_3_ layer. Since the Al_2_O_3_ layer is strongly bound to the nanowire, the crack propagation in the oxide layer can increase the failure rate of the embedded nanowires during cyclic bending. Consequently, the increase in the fractional resistance of the Al_2_O_3_/Ag nanowire is expected to be somewhat higher than that of the bare Ag nanowire electrode. Notwithstanding, the Al_2_O_3_/Ag nanowire electrode still possessed excellent mechanical reliability showing only 8.3% increase in resistance at the end of the bending test of 500,000 cycles and is therefore suitable for high reliability flexible/stretchable applications.

### Durability: Mechanical Wiping Test using IPA

**Figure 6:**
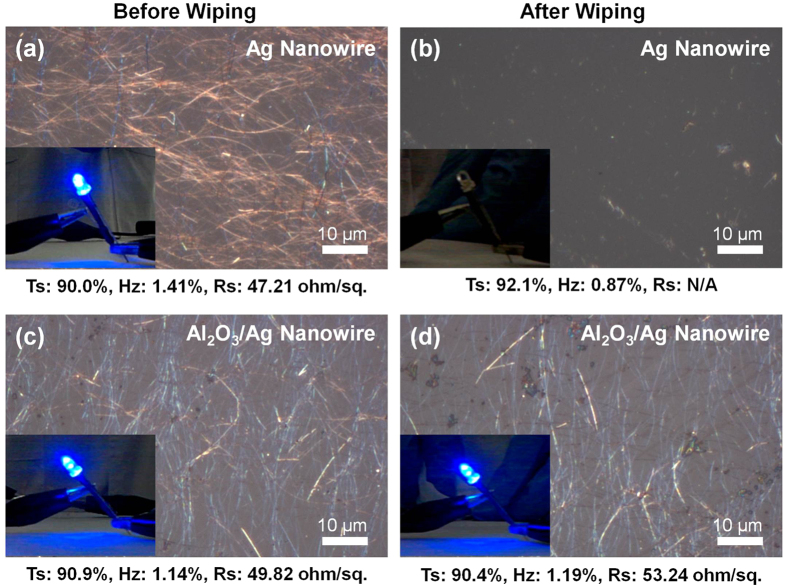
Optical microscopy images of (**a**,**b**) Ag and (**c**,**d**) Al_2_O_3_/Ag nanowire electrodes: (**a**,**c**) and (**b**,**d**) are before and after the wiping test (five times) using IPA, respectively. Here, Ts, Hz, and Rs in each panel represent measured transmittance, haze, and sheet resistance, respectively. Insets are the photographs of LED lights connected to the each specimen.

During the manufacturing process of display and electronic devices, different types of external damages, such as scratch or delamination, can be caused on the electrodes as a result of the harsh processing environment. Therefore, the transparent/flexible electrode needs to be very durable and withstand external damage to ensure the fabrication of highly reliable devices^34^,^35^. A mechanical wiping test using IPA was performed on both bare Ag and Al_2_O_3_/Ag nanowire electrodes on the glass substrates to assess their durability. The test consisted of wiping the electrodes five times while measuring the optical and electrical properties after each wipe. Figure 6 presents the optical microscopy images of both the Ag nanowire electrodes before and after the wiping test (wiping five times). The transmittance, haze, and sheet resistance values before and after the wiping test are also given in Fig. 6; more detailed test results as a function of the number of wipes are also included in Figure S2 in the Supporting information. While the sheet resistance of the bare Ag nanowire electrode increased so greatly that it could no longer be measured after a single IPA wipe, the Al_2_O_3_/Ag nanowire electrode maintained its initial low sheet resistance. The optical microscopy image revealed that the conductivity reduction of the bare Ag nanowire electrode was due to delamination, as shown in Fig. 6a,b. In contrast, the Al_2_O_3_/Ag nanowire electrode showed no significant loss of Ag nanowires even after wiping several times, as shown in Fig. 6c,d. As confirmed from TEM analysis (see Figure S1), the conformal Al_2_O_3_ over-coating was effective in preventing the loss of Ag nanowires during the wiping test by anchoring them on the substrate.

The enhanced durability against mechanical wiping was further demonstrated during the operation of a light-emitting diode (LED) with Ag nanowire electrodes, in which the loss of conductivity decreased the brightness of the LED. The LED with a bare Ag nanowire electrode was completely turned off after just one single wipe (insets of Fig. 6a,b and Supporting Movie 01). In contrast, as shown in the insets of Fig. 6c,d and Supporting Movie 02, no noticeable degradation in brightness was observed for the LED using an Al_2_O_3_/Ag nanowire electrode even after wiping several times with IPA.

## Conclusion

Excellent thermal and ambient stabilities were achieved for Ag nanowire electrodes encapsulated with an ultra-thin Al_2_O_3_ layer (~5.3 nm) coated by low-temperature (100 °C) ALD. The conformal deposition of the high melting Al_2_O_3_ encapsulation layer was able to prevent agglomeration of Ag nanowires by suppressing the surface diffusion of Ag atoms, thereby offering a high thermal stability even when annealed at temperatures up to 380 °C for 100 min. In addition, the Al_2_O_3_ encapsulation layer effectively blocked the diffusion of H_2_O, which significantly enhanced the ambient stability of nanowires to more than 1,080 h at 85 °C under a relative humidity of 85%. With respect to flexibility, cyclic bending test results concluded that Al_2_O_3_/Ag nanowire electrodes had a superior mechanical reliability when compared to conventional ITO films. The ultra-thinness of the Al_2_O_3_ encapsulation layer was able to render the Ag nanowire electrode flexible and improved its reliability. Lastly, Al_2_O_3_ encapsulation provided the Ag nanowire electrode with excellent durability against mechanical wiping using IPA. This was further demonstrated during the functioning of LEDs where no decrease in brightness was observed even after repeated wiping tests. The Al_2_O_3_/Ag nanowire electrodes with high thermal, ambient, and mechanical reliability reported in this study will enlarge the application potential of this material to different types of flexible/transparent devices experiencing harsh fabrication or operation conditions.

## Methods

### Fabrication and characterization of Ag nanowire electrodes

Ag nanowire solution in IPA was provided by BASF Electronic Materials R&D Center, Asia. The average length and diameter of the nanowires were approximately 25 μm and 30 nm, respectively. Doctor-blading was used to coat the Ag nanowires on glass and PET substrates; the blade was fixed at a height of 30 μm above the substrate surface and the speed of the blade movement was 10 mm/s. The as-coated Ag nanowires on the glass and PET substrates were then baked in air at 120 °C for 10 min to completely evaporate the solvent. Transmittance and haze were measured following the ASTM D1003 standard (procedure A) using a UV-Vis spectrometer (haze-gard i) from BYK-Gardner Instrument. A four point probe system (FPP-2400) from Dasol Engineering Co., Ltd., was used to measure the sheet resistance of the Ag nanowire electrodes. The surface morphology of the samples was examined using a field-emission SEM (FE-SEM, Phillips, XL30 ESEM-FEG). A box furnace was used for the thermal stability test in the temperature range from 25 °C to 380 °C. The ambient stability test was conducted in a thermo-hygrostat while maintaining ambient conditions with a relative humidity of 85% at 85 °C.

### ALD of Al_2_O_3_ encapsulation layer

The encapsulation of Ag nanowires with Al_2_O_3_ was carried out using the ALD process with TMA and H_2_O precursors and a substrate temperature of 100 °C. Each ALD cycle for the deposition of Al_2_O_3_ consisted of the consecutive injection of TMA/N_2_/H_2_O/N_2_ gases.

### Cyclic bending test

The cyclic bending test was carried out using a bending fatigue tester capable of providing a consistent bending strain for more than 500,000 cycles while measuring the resistance with a resolution of ~0.003 ohm^28^. PET substrates coated with Ag or Al_2_O_3_/Ag nanowire electrodes were fixed on two parallel plates using metal bolts and Cu pads in each plate were contacted to the specimen for the measurement of resistance during cyclic bending. The lower plate moved horizontally with a fixed distance of 10 mm and a bending speed of 300 cycles/min to apply a repeatable bending strain. The strain imposed on the specimen was calculated to be 2.5% using the equation ε = y/R, where y is the distance from neutral plane and R is the bending radius (one half of the gap between the plates, 2.5 mm). The bending fatigue test was conducted more than 3 times for each specimen under identical test conditions to ensure the reproducibility of the results. A more detailed description of the bending fatigue tester can be found in ref. 28.

## Additional Information

**How to cite this article:** Hwang, B. *et al*. Highly Flexible and Transparent Ag Nanowire Electrode Encapsulated with Ultra-Thin Al_2_O_3_: Thermal, Ambient, and Mechanical Stabilities. *Sci. Rep.* **7**, 41336; doi: 10.1038/srep41336 (2017).

**Publisher's note:** Springer Nature remains neutral with regard to jurisdictional claims in published maps and institutional affiliations.

## Supplementary Material

Supplementary Information

Supporting Movie 1

Supporting Movie 2
