# Supplementary material for: Highly Flexible and Transparent Ag Nanowire Electrode Encapsulated with Ultra-Thin Al2O3: Thermal, Ambient, and Mechanical Stabilities
Source: Sci Rep. 2017 Jan 27;7:41336. doi: 10.1038/srep41336 (PMC5269670; doi:10.1038/srep41336)
Supplement: Supplementary Information [file srep41336-s1.doc]

**Supporting Information for “Highly Flexible and Transparent Ag Nanowire Electrode Encapsulated with Ultra-Thin Al_2_O_3_: Thermal, Ambient, and Mechanical Stabilities”**

**Byungil Hwang^1†^, Youngseo An^2†^, Hyangsook Lee^2,3^, Eunha Lee^3^, Stefan Becker^1^,**

**Yong-Hoon Kim^2,4^, and Hyoungsub Kim^2^***

^1^ BASF Electronic Materials R&D Center Asia, Suwon, 16419, Republic of Korea

^2^ School of Advanced Materials Science and Engineering, Sungkyunkwan University, Suwon, 16419, Republic of Korea

^3^Analytical Engineering Group, Samsung Advanced Institute of Technology (SAIT), Samsung Electronics Co., Suwon 16678, Republic of Korea

^4^ SKKU Advanced Institute of Nanotechnology (SAINT), Sungkyunkwan University, Suwon, 16419, Republic of Korea

* hsubkim@skku.edu

**Table S1.** Comparison of our experimental results with previous reports.

| **Reference** | **Coating material** | **Method** | **Transmittance** | **Thermal stability** | **Ambient stability** | **Mechanical reliability (bending test)** | **Durability (wiping test)** |
| --- | --- | --- | --- | --- | --- | --- | --- |
| Song et al. (s1) | TiO_2_ | Sol-gel | ~86% | 300 °C,  60 min | N/A | 500 cycles | N/A |
| Hwang et al.  (s2) | Reduced graphene oxide | Spray | ~91% | N/A | 70 °C,  132 hr  (air) | 800,000 cycles | N/A |
| Kim et al.  (s3) | ZnO | Sputter | ~92% | 300 °C | N/A | 500 cycles | N/A |
| Chen et al.  (s4) | ZnO | ALD | ~91% | 300 °C,  60 hr | N/A | N/A | N/A |
| Duan et al.  (s5) | ZnO | ALD | ~80% | N/A | N/A | N/A | N/A |
| Ali et al.  (s6) | Al_2_O_3_ | ALD | N/A | N/A | N/A | N/A | N/A |
| This work | Al_2_O_3_ | ALD | ~91% | 380 °C, 100 min | 85 °C,  1,080 hr  (RH 85%) | 500,000 cycles | 5 times of wipe |

**Fig. S1.** (a) Cross-sectional TEM image of the Al_2_O_3_/Ag electrode. (b) High-angle annular dark field (HAADF) and energy-dispersive X-ray spectroscopy (EDS) mapping images of the Al_2_O_3_/Ag electrode (the scale bar is 50 nm). The ALD-Al_2_O_3_ layer with a thickess of ~5.3 nm conformally covered the Ag nanowires. The EDS analysis confirms that the polymeric residues from the Ag nanowire ink remained beneath the Ag nanowire after the coating of the Ag nanowires.

**Fig. S2.** Changes in transmittance, haze, and sheet resistance as a function of the number of IPA wipes: (a) bare Ag and (b) Al_2_O_3_/Ag nanowire electrodes.

**Movie Legends:**

Please see also the detail in the main text.

**Supporting movie S1**. Operation of a LED connected with a bare Ag nanowire electrode during the wiping test using IPA.

**Supporting movie S2**. Operation of a LED connected with a Al_2_O_3_/Ag nanowire electrode during the wiping test using IPA.

**References**

(s1) Song, T. B. *et al.*, Highly robust silver nanowire network for transparent electrode. *ACS Appl. Mater. Interfaces.* **7**, 24601-7 (2015)

(s2) Hwang, B., Park, M., Kim, T. & Han, S. M., Effect of RGO deposition on chemical and mechanical reliability of Ag nanowire flexible transparent electrode. *RSC Adv.* **6**, 67389-67395 (2016).

(s3) Kim, A., Won, Y., Woo, K., Kim, C.-H. & Moon, J., Highly transparent low resistance Zno/Ag nanowire/Zno composite electrode for thin film solar cells. *ACS Nano.* **7**, 1081-1091 (2013). (s4) Chen, D. *et al.*, Thermally stable silver nanowire-polyimide transparent electrode based on atomic layer deposition of zinc oxide on silver nanowires. *Adv. Funct. Mater.* **25**, 7512-7520 (2015).

(s5) Duan, Y. H. *et al.*, High-performance flexible ag nanowire electrode with low-temperature atomic-layer-deposition fabrication of conductive-bridging Zno film. *Nanoscale Res. Lett.* **10**, 90 (2015).

(s6) Ali, K., Duraisamy, N., Kim, C. Y. & Choi, K.-H., Al_2_O_3_ coatings fabrication on silver nanowires through low temperature atomic layer deposition. *Mater. Manuf. Process.* **29**, 1056-1061 (2014).
